# Supplementary material for: Extensive standing genetic variation from a small number of founders enables rapid adaptation in Daphnia
Source: Nat Commun. 2021 Jul 14;12:4306. doi: 10.1038/s41467-021-24581-z (PMC8280168; doi:10.1038/s41467-021-24581-z)
Supplement: Supplementary file 1 — Supplementary Information [file 41467_2021_24581_MOESM1_ESM.pdf]

Supplementary Information for

Extensive standing genetic variation from a small number of founders enables rapid adaptation in *Daphnia*

Anurag Chaturvedi[1,2,3*†], Jiarui Zhou[3,4†], Joost A. M. Raeymaekers[5,6], Till Czypionka[1], Luisa Orsini[3], Craig E. Jackson[7], Katina I. Spanier[1], Joseph R. Shaw[7], John K. Colbourne[3], Luc De Meester[1,8,9].

[1] *Laboratory of Aquatic Ecology, Evolution and Conservation, KU Leuven, Ch. de Bériotstraat 32, 3000 Leuven, Belgium.*
[2] *Department of Ecology and Evolution, University of Lausanne, Biophore Building, 1015, Lausanne, Switzerland*
[3] *Environmental Genomics Group, School of Biosciences, University of Birmingham, Birmingham B15 2TT, UK*
[4] *Centre for Computational Biology, University of Birmingham, Birmingham B15 2TT, UK*
[5] *Laboratory of Biodiversity and Evolutionary Genomics, KU Leuven, Ch. de Bériotstraat 32, 3000 Leuven, Belgium.*
[6] *Faculty of Bioscience and Aquaculture, Nord University, Universitetsalléen 11, N-8026, Bodø, Norway*
[7] *O'Neill School of Public and Environmental Affairs, Indiana University, Bloomington, Indiana 47405, USA*
[8] *Leibniz Institut für Gewässerökologie und Binnenfischerei (IGB), Müggelseedamm 310, 12587 Berlin, Germany*
[9] *Institute of Biology, Freie Universität Berlin, Königin-Luise-Strasse 1-3, 14195 Berlin, Germany*

*Correspondence to: anurag.chaturvedi@unil.ch

† AC, JZ contributed equally to this work

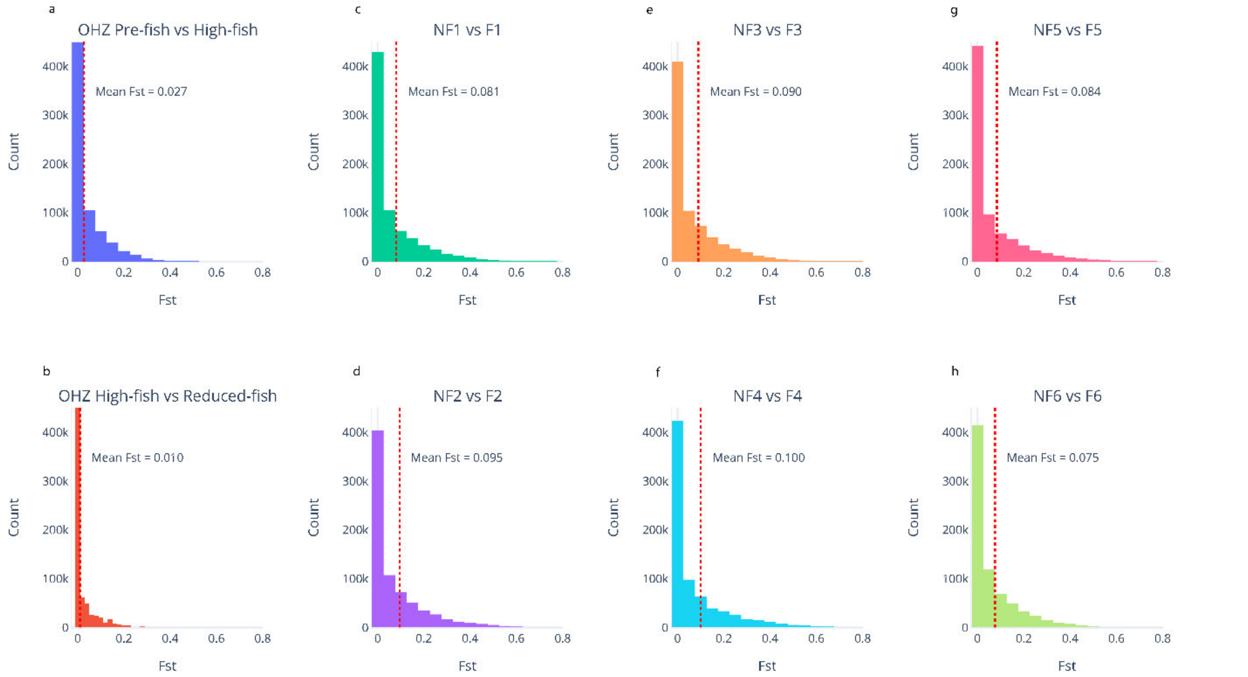

Supplementary Figure 1.

Observed genetic differentiation (F_{ST}) of *Daphnia magna* temporal subpopulations separated by time in OHZ pond (a-b) and of six population pairs contrasting a population with and without fish (c-h). Distribution of the observed genetic differentiation quantified as F_{ST} values across all SNPs in the genome for **(a)** the pre-fish to high-fish transition, **(b)** the high-fish to reduced-fish transition, **(c)** the population pair contrasting fishless pond DANA with fish pond ZW4 (NF1 vs F1), **(d)** the population pair contrasting fishless pond U2 with fish pond LRV (NF2 vs F2), **(e)** the population pair contrasting fishless pond TER1 with fish pond ZW3 (NF3 vs F3), **(f)** the population pair contrasting fishless pond MO with fish pond OHN (NF4 vs F4), **(g)** the population pair contrasting fishless pond KNO15 with fish pond OM2 (NF5 vs F5), and **(h)** the population pair contrasting fishless pond TER2 with fish pond OM3 (NF6 vs F6).

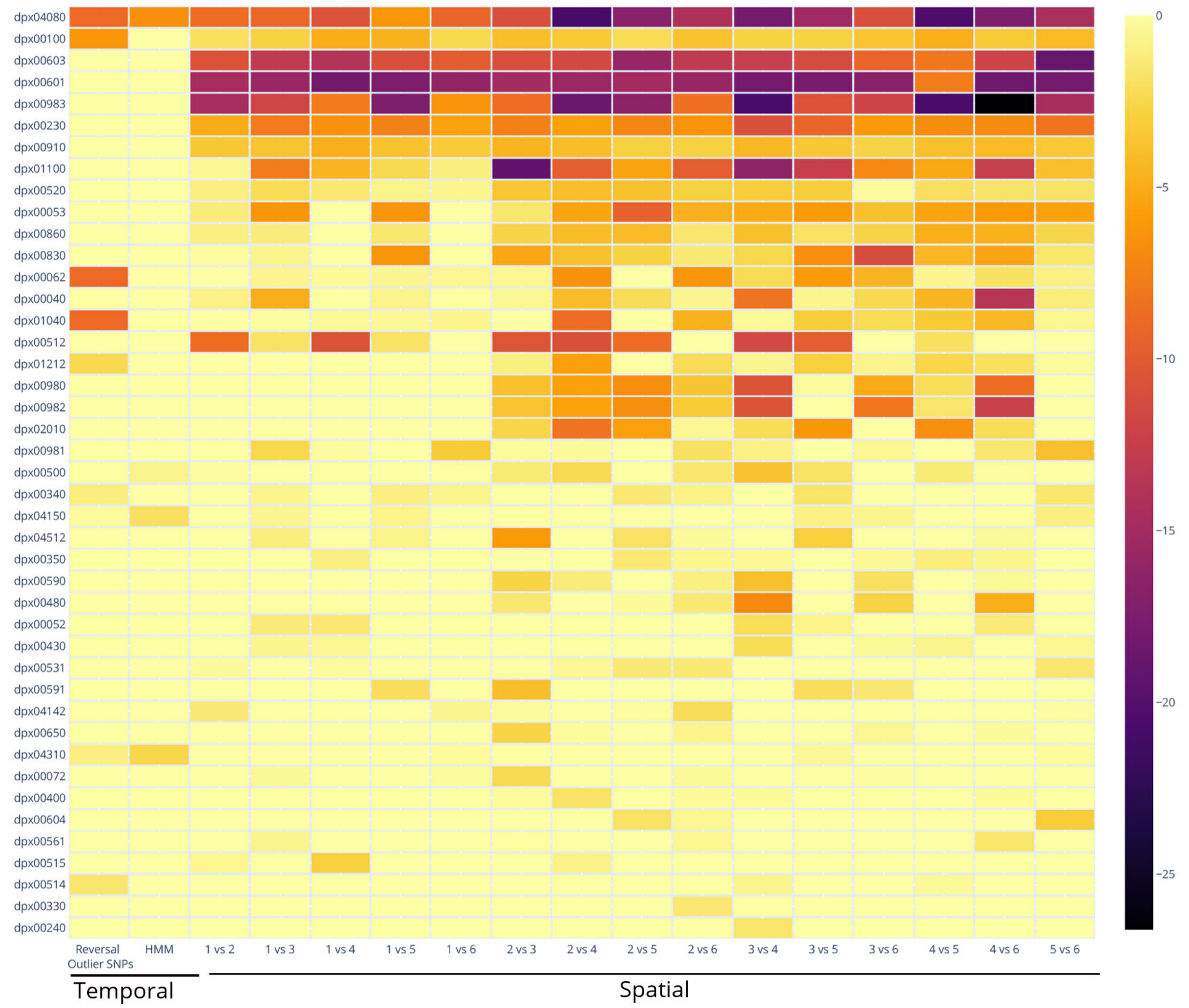

KEGG ID	Pathway name	KEGG ID	Pathway name	KEGG ID	Pathway name
dpx04080	Neuroactive ligand-receptor interaction	dpx00512	Mucin type O-glycan biosynthesis	dpx00531	Glycosaminoglycan degradation
dpx00100	Steroid biosynthesis	dpx01212	Fatty acid metabolism	dpx00591	Linoleic acid metabolism
dpx00603	Glycosphingolipid biosynthesis - globo and isoglobo series	dpx00980	Metabolism of xenobiotics by cytochrome P450	dpx04142	Lysosome
dpx00601	Glycosphingolipid biosynthesis - lacto and neolacto series	dpx00982	Drug metabolism - cytochrome P450	dpx00650	Butanoate metabolism
dpx00983	Drug metabolism - other enzymes	dpx02010	ABC transporters	dpx04310	Wnt signaling pathway
dpx00230	Purine metabolism	dpx00981	Insect hormone biosynthesis	dpx00072	Synthesis and degradation of ketone bodies
dpx00910	Nitrogen metabolism	dpx00500	Starch and sucrose metabolism	dpx00400	Phenylalanine, tyrosine and tryptophan biosynthesis
dpx01100	Metabolic pathways	dpx00340	Histidine metabolism	dpx00604	Glycosphingolipid biosynthesis - ganglio series
dpx00520	Amino sugar and nucleotide sugar metabolism	dpx04150	mTOR signaling pathway	dpx00561	Glycerolipid metabolism
dpx00053	Ascorbate and aldarate metabolism	dpx04512	ECM-receptor interaction	dpx00515	Mannose type O-glycan biosynthesis
dpx00860	Porphyrin and chlorophyll metabolism	dpx00350	Tyrosine metabolism	dpx00514	Other types of O-glycan biosynthesis
dpx00830	Retinol metabolism	dpx00590	Arachidonic acid metabolism	dpx00330	Arginine and proline metabolism
dpx00062	Fatty acid elongation	dpx00480	Glutathione metabolism	dpx00240	Pyrimidine metabolism
dpx00040	Pentose and glucuronate interconversions	dpx00052	Galactose metabolism		
dpx01040	Biosynthesis of unsaturated fatty acids	dpx00430	Taurine and hypotaurine metabolism		

Supplementary Figure 2.

KEGG pathway enrichment for temporal and spatial comparisons. Enriched KEGG pathways (Fisher's exact test, one sided, upper tail with FDR correction, P value <0.05) for genes that show shared divergence in allele frequencies of SNPs during both transitions in time (temporal,

resurrection genomics on population OHZ) or show shared divergence in all possible combinations of population pairs in the spatial dataset. For the resurrection genomic analysis, the analysis has both been done for SNPs that behave as an outlier in both transitions ("Reversal outlier SNPs") and for SNPs that during both transitions are identified to be located in a genomic island of high divergence categorized by HMM ("HMM"). For spatial comparisons, a number in the label coding refers to the number of the population pair (e.g. "1 vs 2" shows the KEGG enrichment for shared islands of divergence between the contrast between populations NF1 and F1 and the contrast between populations NF2 and F2). The color coding indicates log transformed adjusted p-values. The table provides the KEGG pathway IDs and the KEGG pathway names in the order as they appear in the figure (i.e. decreasing average significance).

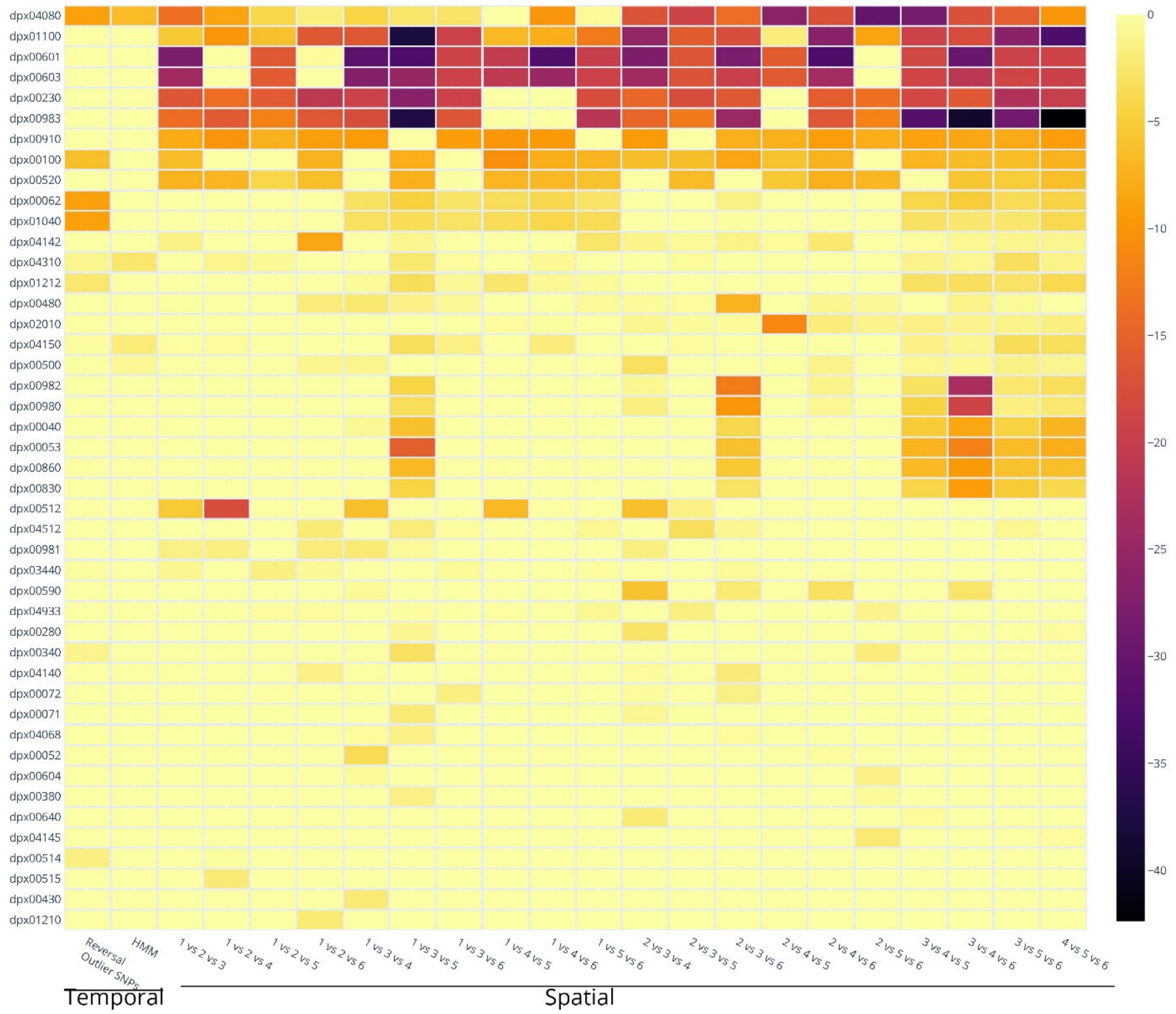

KEGG ID	Pathway name	KEGG ID	Pathway name	KEGG ID	Pathway name
dpx04080	Neuroactive ligand-receptor interaction	dpx02010	ABC transporters	dpx00280	Valine, leucine and isoleucine degradation
dpx01100	Metabolic pathways	dpx04150	mTOR signaling pathway	dpx00340	Histidine metabolism
dpx00601	Glycosphingolipid biosynthesis - lacto and neolacto series	dpx00500	Starch and sucrose metabolism	dpx04140	Autophagy - animal
dpx00603	Glycosphingolipid biosynthesis - globo and isoglobo series	dpx00982	Drug metabolism - cytochrome P450	dpx00072	Synthesis and degradation of ketone bodies
dpx00230	Purine metabolism	dpx00980	Metabolism of xenobiotics by cytochrome P450	dpx00071	Fatty acid degradation
dpx00983	Drug metabolism - other enzymes	dpx00040	Pentose and glucuronate interconversions	dpx04068	FoxO signaling pathway
dpx00910	Nitrogen metabolism	dpx00053	Ascorbate and aldarate metabolism	dpx00052	Galactose metabolism
dpx00100	Steroid biosynthesis	dpx00860	Porphyrin and chlorophyll metabolism	dpx00604	Glycosphingolipid biosynthesis - ganglio series
dpx00520	Amino sugar and nucleotide sugar metabolism	dpx00830	Retinol metabolism	dpx00380	Tryptophan metabolism
dpx00062	Fatty acid elongation	dpx00512	Mucin type O-glycan biosynthesis	dpx00640	Propanoate metabolism
dpx01040	Biosynthesis of unsaturated fatty acids	dpx04512	ECM-receptor interaction	dpx04145	Phagosome
dpx04142	Lysosome	dpx00981	Insect hormone biosynthesis	dpx00514	Other types of O-glycan biosynthesis
dpx04310	Wnt signaling pathway	dpx03440	Homologous recombination	dpx00515	Mannose type O-glycan biosynthesis
dpx01212	Fatty acid metabolism	dpx00590	Arachidonic acid metabolism	dpx00430	Taurine and hypotaurine metabolism
dpx00480	Glutathione metabolism	dpx04933	AGE-RAGE signaling pathway in diabetic complications	dpx01210	2-Oxocarboxylic acid metabolism

Supplementary Figure 3.

KEGG pathway enrichment for temporal and spatial comparisons. Enriched KEGG pathways (Fisher's exact test, one sided, upper tail with FDR correction, P value <0.05) for genes that show

shared divergence in allele frequencies of SNPs during both transitions in time (temporal, resurrection genomics on population OHZ) or show shared divergence in all possible combinations of three population pairs in the spatial dataset. For the resurrection genomic analysis, the analysis has both been done for SNPs that behave as an outlier in both transitions ("Reversal outlier SNPs") and for SNPs that during both transitions are identified to be located in a genomic island of high divergence categorized by HMM ("HMM"). For spatial comparisons, a number in the label coding refers to the number of the population triplet (e.g. "1 vs 2 vs 3" shows the KEGG enrichment for shared islands of divergence between the three contrasts: between populations NF1 and F1, between populations NF2 and F2, and between populations NF3 and F3). The color coding indicates log transformed adjusted p-values. The table provides the KEGG pathway IDs and the KEGG pathway names in the order as they appear in the figure (i.e. decreasing average significance).

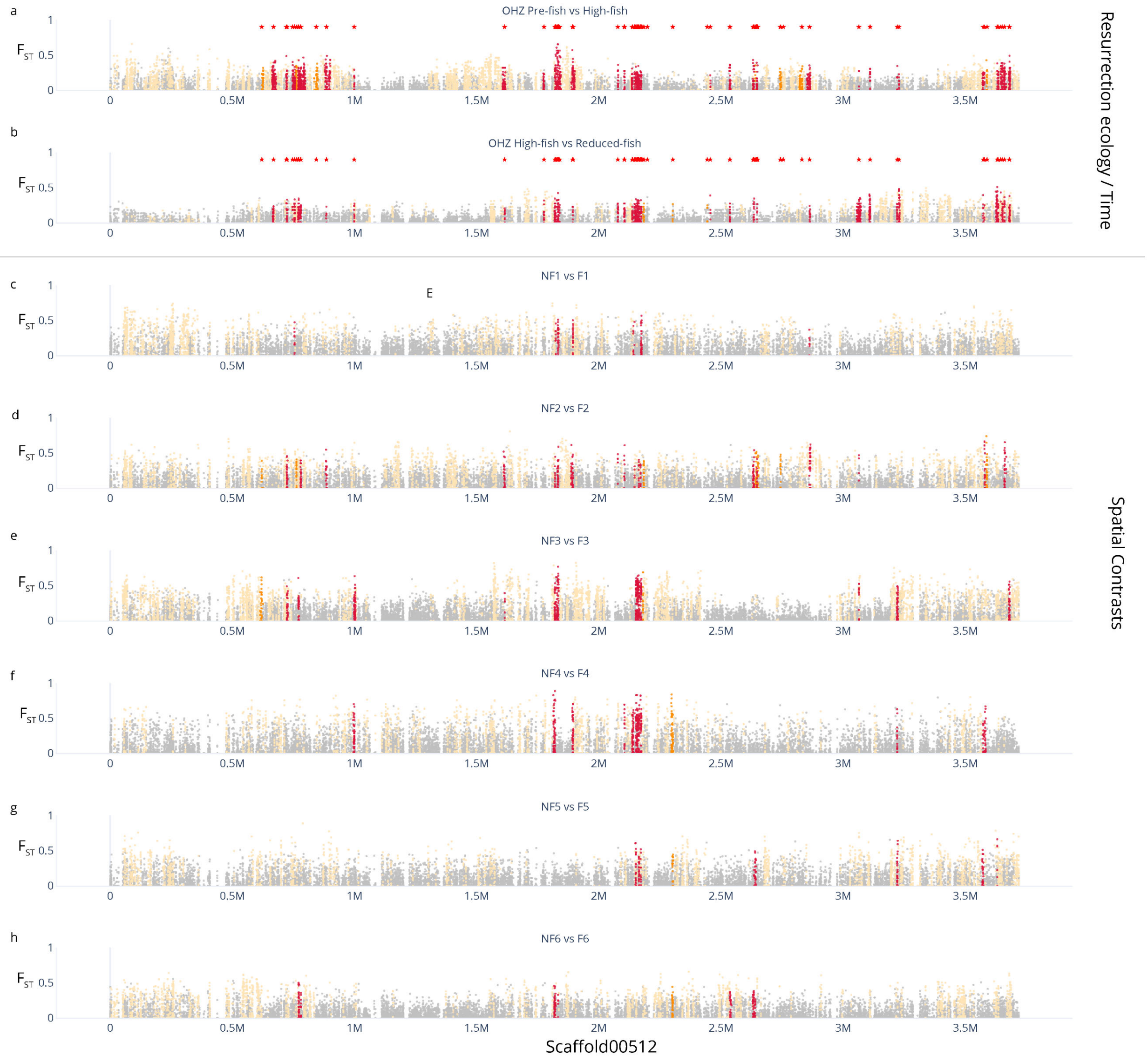

Supplementary Figure 4.

Genomic islands of high divergence as identified by a Hidden Markov Model in scaffold00512.

Hidden Markov Model SNP categories representing genomic regions of high genetic differentiation between *Daphnia magna* temporal subpopulations separated by time (**a**: OHZ pre-fish to high-fish transition; **b**: OHZ high-fish to reduced-fish transition) and six independent contrasts between populations inhabiting habitats with and without fish in a spatial survey, for the largest scaffold of the *D. magn*a genome assembly scaffold00512 (3718170 bp). The red dots indicate SNPs that are identified by the HMM to lie within a largely overlapping island of divergence in the two temporal transitions. Red dots in the spatial contrasts indicate SNPs that are within islands of divergence shared by the two transitions in population OHZ. Yellow dots in the temporal populations (**a-b**)

are islands of divergence that are not shared across transitions. Yellow dots in the spatial contrasts (**c-h**) indicate SNPs that are within islands of divergence that overlap with islands of one of the two transitions in OHZ. Red stars in plots **a-b** indicates outlier SNPs.

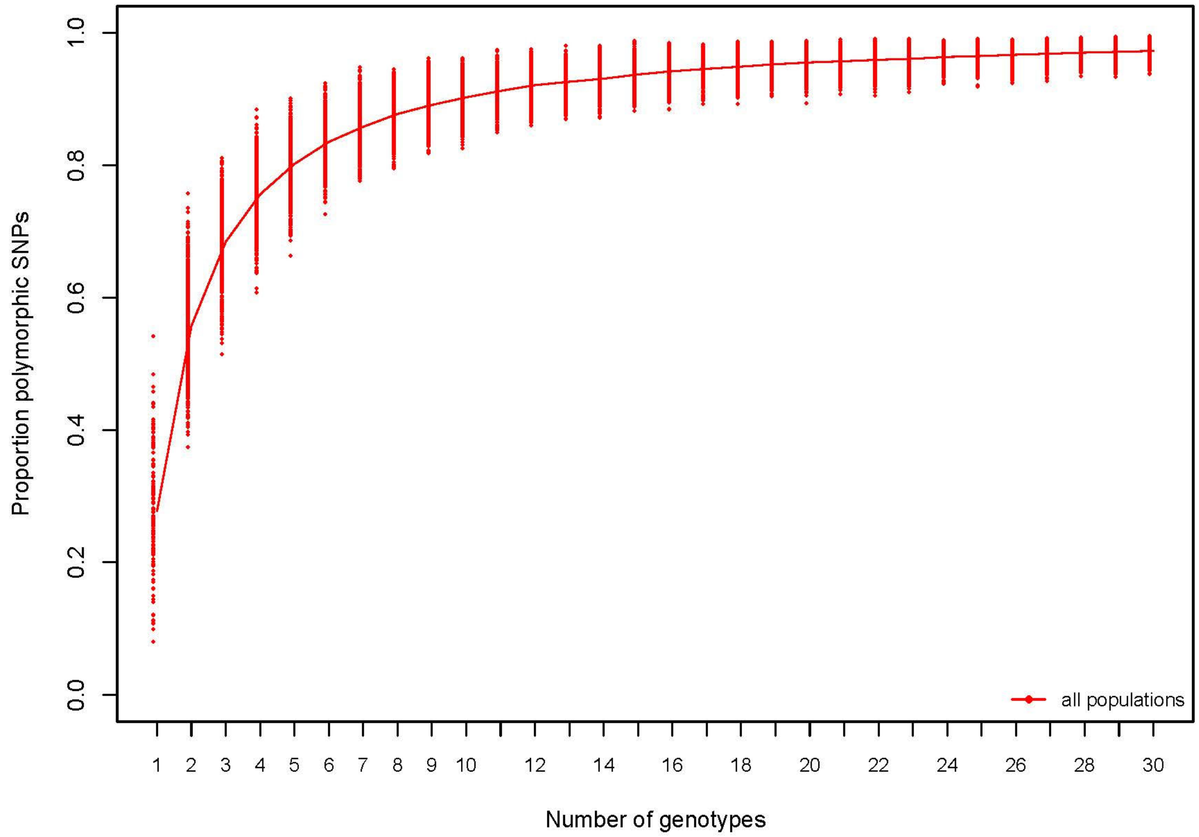

Supplementary Figure 5.

The proportion of SNPs that were potentially under positive selection (i.e. excluding hitchhiked SNPs) in the OHZ population and that were found in the spatial dataset (N = 1003; i.e. 90.4% of the SNPs from Fig. 4a) that is polymorphic as a function of an increasing number of individual genotypes from the 12 sampled spatial populations (i.e six populations from ponds without fish (NF) and six populations from ponds with fish (F)) (solid, thick red line).

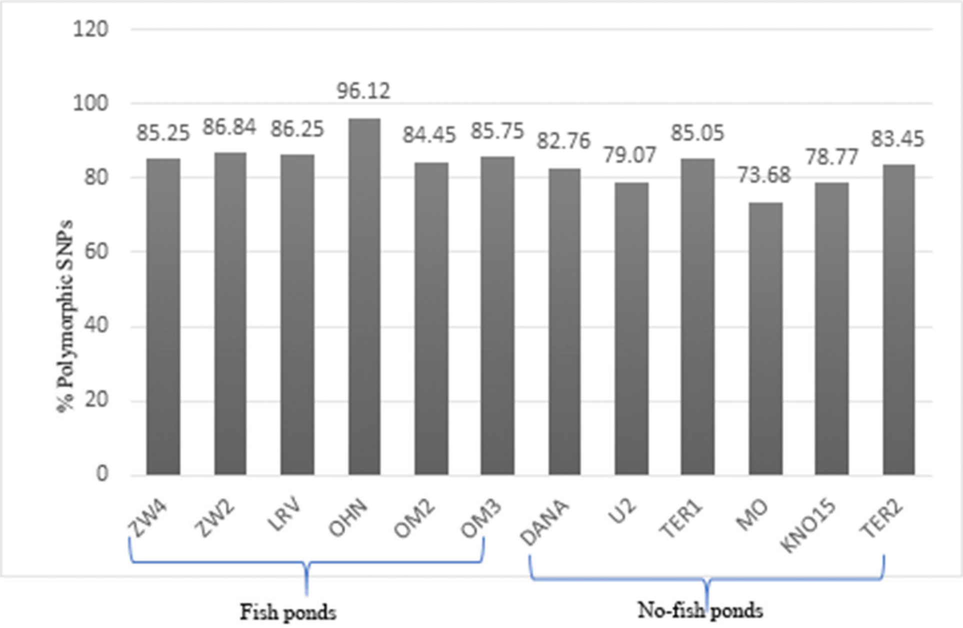

Supplementary Figure 6.

The proportion of SNPs that are polymorphic in each pond from the 12 sampled spatial populations (i.e. six populations from ponds without fish (NF) and six populations from ponds with fish (F)) and that were potentially under positive selection (i.e. excluding hitchhiked SNPs) in the OHZ population (analysis done on SNPs that were also present in the spatial dataset; N = 1003; i.e. 90.4 % of the total number of SNPs that were potentially under positive selection (see Fig. 4a)).

Supplementary Table 1.

The sampled *D. magna* populations (in time and space) and their characteristics in terms of presence or absence of fish and number of whole genome re-sequenced genotypes per population.

Population setting	Population	No. of genotyped individuals	Location	Lat	Long	Stressor*
Temporal	OHZ	12	Oud-Heverlee Zuid (inland)	50° 50´	4° 39’	Pre-fish
Temporal	OHZ	12	Oud-Heverlee Zuid (inland)	50° 50´	4° 39’	High-fish
Temporal	OHZ	12	Oud-Heverlee Zuid (inland)	50° 50´	4° 39’	Reduced-fish
Spatial	DANA (NF1)	11	Damme (coast)	51° 15'	03° 16'	NF
Spatial	U2 (NF2)	12	Uitkerke (coast)	51° 16'	03° 05'	NF
Spatial	TER1 (NF3)	17	Neerijse (inland)	50° 49'	04° 35'	NF
Spatial	MO (NF4)	8	Moorsel (inland)	50° 51'	04° 32'	NF
Spatial	KNO15 (NF5)	10	Knokke (coast)	51° 20'	03° 20'	NF
Spatial	TER2 (NF6)	15	Neerijse (inland)	50° 49'	04° 36'	NF
Spatial	ZW4 (F1)	11	Oud-Heverlee (inland)	50° 49'	04° 39'	F
Spatial	LRV (F2)	12	Oud-Heverlee (inland)	50° 49'	04° 38'	F
Spatial	ZW3 (F3)	14	Oud-Heverlee (inland)	50° 49'	04° 39'	F
Spatial	OHN (F4)	12	Oud-Heverlee (inland)	50° 50'	04° 39'	F
Spatial	OM2 (F5)	10	Heverlee (inland)	50° 51'	04° 43'	F
Spatial	OM3 (F6)	12	Heverlee (inland)	50° 51'	04° 43'	F

* NF=Fishless pond (“No Fish”); F=Fish pond

Supplementary Table 2.

Estimated effective population size (Ne) of the sampled *D. magna* populations.

Population setting	Population*	Effective population size (Ne)
Temporal	OHZ pre-fish	1.66E+06
Temporal	OHZ high-fish	1.72E+06
Temporal	OHZ reduced-fish	1.72E+06
Spatial	DANA (NF1)	1.36E+06
Spatial	U2 (NF2)	1.25E+06
Spatial	TER1 (NF3)	1.11E+06
Spatial	MO (NF4)	1.39E+06
Spatial	KNO15 (NF5)	1.43E+06
Spatial	TER2 (NF6)	1.17E+06
Spatial	ZW4 (F1)	1.34E+06
Spatial	LRV (F2)	1.06E+06
Spatial	ZW3 (F3)	1.10E+06
Spatial	OHN (F4)	1.31E+06
Spatial	OM2 (F5)	1.45E+06
Spatial	OM3 (F6)	1.36E+06

* NF=Fishless pond ("No Fish"); F=Fish pond

Supplementary Table 3.
Characteristics of high differentiation regions in two temporal and six independent spatial replicates of fish / no-fish contrasts based on a Hidden markov model (HMM) analysis. HGD: high genetic differentiation. For codes of NF1 and F1 see Table S1.

Population setting	Population comparison*	Number of HGD SNPs in islands	Total number of islands	Mean Fst/island	Mean SNPs/island	Std SNPs/island	Mean island lengths (bp)
Temporal	OHZ pre-fish vs high-fish	128969	6111	0.11	21.10	28.72	2428
Temporal	OHZ high-fish vs reduced-fish	49148	2879	0.10	17.07	19.49	1713
Spatial	NF1 vs F1	90624	6369	0.20	14.23	14.76	1879
Spatial	NF2 vs F2	141983	7493	0.20	18.95	24.45	2506
Spatial	NF3 vs F3	112526	6962	0.20	16.16	17.74	2053
Spatial	NF4 vs F4	99725	4136	0.22	24.11	28.21	3290
Spatial	NF5 vs F5	103241	5322	0.20	19.40	20.96	2564
Spatial	NF6 vs F6	109483	6723	0.18	16.28	17.48	2161

*NF=Fishless pond (“No Fish”); F=Fish pond

Supplementary text

Comparison of genomic variation to genetic trait changes in the population inhabiting OHZ

A synopsis of Stoks et al. (2016)[1], a quantitative genetic study of trait variation in the OHZ population

For the same population as studied here in the temporal analysis (i.e. the population inhabiting pond OHZ), and actually for the same set of 36 clones, evolution in a total of 14 different life history and behavioural traits was quantified in a common garden experiment. These data are published (Stoks et al 2016, Ecology Letters[1]). In this study, all genotypes were cultured for two generations under common garden conditions prior to quantify trait value so as to obviate interference from maternal effects. The resulting trait values thus represent genotypic trait values. The results of that study are summarized in Figure 1b as mean changes in trait values of the three temporal subpopulations separated in time. This figure emphasizes the reversal in mean trait values, but Stoks et al. (2016)[1] shows and includes analyses of variation in the mean trait values and responses to fish kairomones among clones.

In Stoks et al. (2016)[1], trait values were quantified both in the absence and presence of fish kairomones (medium conditioned by the presence of fish). *Daphnia* can recognize the presence of fish chemically and can show adaptive plasticity responses. By quantifying trait values in both the absence and presence of fish kairomones, Stoks et al (2016)[1] quantified the reaction norms across this change in predation risk by fish for 14 traits of 36 clones (12 clones for each of 3 temporal subpopulations separated in time). Out of 14 traits studied, 13 traits showed significant evolutionary changes, and these changes were in line with expectations under the assumption of adaptive evolution. For example, in the case of diel vertical migration measured as phototactic behaviour, adaptive evolution would involve a change towards becoming more negatively phototactic, as residing deeper in the water column is a well-documented and effective defence against predation by visually hunting fish. This is also what we observed: during the first transition from pre-fish to high-fish, there was a significant evolution of phenotypic plasticity. More specifically, in the high-fish predation temporal subpopulation most clones show a strong and significant phenotypic plasticity towards more negative phototaxis (i.e., safer behaviour) in the presence of fish smell, whereas most clones in the pre-fish temporal subpopulation did not show

such a phenotypic plasticity response. This thus reflects adaptive evolution through genetic changes in phenotypic plasticity. In the second transition, from high to reduced fish predation, we did not see an evolution of phenotypic plasticity, but rather observed a genetic shift in mean trait values to less negatively phototactic behaviour. So the clones remain responsive to fish but in general behave less safe - in line with expectations stemming from the fact that there were still fish present in the habitat, but in less high abundances than before (cf. "reduced-fish" versus "high-fish"). Similar adaptive changes were observed for size at maturity, with animals genetically becoming smaller in the high-fish temporal subpopulation and partly reversing that response to become somewhat larger again in the reduced-fish temporal subpopulation. In summary Stoks et al[2] observed adaptive evolution in multiple traits and that these trait changes were (partly) reversible.

A comparison of patterns in genotypic trait values and genome-wide allele frequency changes

Our set of 36 clones is insufficient to perform a reliable Genome-Wide Association Study (GWAS[2,3]). Yet, there are important similarities between the genotypic trait changes observed by Stoks et al. (2016) and the genome-wide changes observed in the current study. Most notably, there is a striking parallel in the occurrence of reversals. In the current study we observed reversals in 99% of SNPs (1753 SNPs out of 1771) that show significant changes in allele frequency in both the pre-fish to high-fish and the high-fish to reduced-fish transitions. This percentage of overall reversal is more than expected by chance based on a permutation test (p-value < 1e-4; following the approach described in "Section S6" of Buffalo & Coop (2020)[4]. Such reversals are also observed for the genotypic trait changes for most life history and behavioural traits studied by Stoks et al. (2016)[1] (see Fig. 1b). This dominance of reversals strongly suggests that fish predation pressure (from no to high fish densities in the first transition, from high to reduced fish densities in the second transition) is a key driver of evolution in this system. This is further supported by the observation that the genotypic trait changes observed by Stoks et al. (2016)[1] are in line with the responses one would expect in the scenario of adaptive evolution in response to changes in fish predation pressure. The genomic data also are suggestive of a clear link to functional responses, as genes showing significant changes in allele frequencies were enriched for a limited number of pathways, and all three significantly overrepresented pathways are in the pathway class of

"Environmental Information Processing; Signaling molecules and interaction", and were validated by the spatial analysis.

Supplementary references

1 Stoks, R., Govaert, L., Pauwels, K., Jansen, B. & De Meester, L. Resurrecting complexity: the interplay of plasticity and rapid evolution in the multiple trait response to strong changes in predation pressure in the water flea Daphnia magna. *Ecol Lett* **19**, 180-190, (2016).

2 Korte, A. & Farlow, A. The advantages and limitations of trait analysis with GWAS: a review. *Plant Methods* **9**, 29, (2013).

3 Hong, E. P. & Park, J. W. Sample size and statistical power calculation in genetic association studies. *Genomics Inform* **10**, 117-122, (2012).

4 Buffalo, V. & Coop, G. Estimating the genome-wide contribution of selection to temporal allele frequency change. *Proc Natl Acad Sci U S A* **117**, 20672-20680, (2020).
