# Supplementary material for: Extensive standing genetic variation from a small number of founders enables rapid adaptation in Daphnia
Source: Nat Commun. 2021 Jul 14;12:4306. doi: 10.1038/s41467-021-24581-z (PMC8280168; doi:10.1038/s41467-021-24581-z)
Supplement: Supplementary file 3 — Reporting Summary [file 41467_2021_24581_MOESM3_ESM.pdf]

nature research

Corresponding author(s): Chaturvedi Anurag

Last updated by author(s): Jun 10, 2021

Reporting Summary

Nature Research wishes to improve the reproducibility of the work that we publish. This form provides structure for consistency and transparency in reporting. For further information on Nature Research policies, see our Editorial Policies and the Editorial Policy Checklist.

Statistics

For all statistical analyses, confirm that the following items are present in the figure legend, table legend, main text, or Methods section.

n/a	Confirmed	
☐	☒	The exact sample size (*n*) for each experimental group/condition, given as a discrete number and unit of measurement
☒	☐	A statement on whether measurements were taken from distinct samples or whether the same sample was measured repeatedly
☐	☒	The statistical test(s) used AND whether they are one- or two-sided<br>*Only common tests should be described solely by name; describe more complex techniques in the Methods section.*
☒	☐	A description of all covariates tested
☐	☒	A description of any assumptions or corrections, such as tests of normality and adjustment for multiple comparisons
☐	☒	A full description of the statistical parameters including central tendency (e.g. means) or other basic estimates (e.g. regression coefficient) AND variation (e.g. standard deviation) or associated estimates of uncertainty (e.g. confidence intervals)
☐	☒	For null hypothesis testing, the test statistic (e.g. *F*, *t*, *r*) with confidence intervals, effect sizes, degrees of freedom and *P* value noted<br>*Give P values as exact values whenever suitable.*
☒	☐	For Bayesian analysis, information on the choice of priors and Markov chain Monte Carlo settings
☒	☐	For hierarchical and complex designs, identification of the appropriate level for tests and full reporting of outcomes
☒	☐	Estimates of effect sizes (e.g. Cohen's *d*, Pearson's *r*), indicating how they were calculated

Our web collection on statistics for biologists contains articles on many of the points above.

Software and code

Policy information about availability of computer code

Data collection

Sequencing data was generated using Illumina HiSeq2500 platform for paired-end sequencing with 100 bp read length following manufacturer's instructions. No software was used for data collection.

Data analysis

Population genetic data analysis in this study was performed using the following publicly available programs:
1) FASTQC Version 0.11.6
2) Trimmomatic Version 0.39
3) Bowtie2 Version 2.1.0
4) Picard tools MarkDuplicates Version 1.100(1571)
5) freeBayes v0.9.20-16-g3e35e72
6) VCFtools v0.1.12b
7) diversity R package version 1.9.90
8) vcflib v1.0.1
9) ANGSD v0.930/0.931
10) R package HiddenMarkov v1.8-11
11) Python package NumPy v1.17.4
12) Python package SciPy v1.4.1
13) Python package statsmodels v0.11.1
14) Python package plotly v4.8.1
15) TAFT v2.3

For manuscripts utilizing custom algorithms or software that are central to the research but not yet described in published literature, software must be made available to editors and reviewers. We strongly encourage code deposition in a community repository (e.g. GitHub). See the Nature Research guidelines for submitting code & software for further information.

Data

Policy information about availability of data

All manuscripts must include a data availability statement. This statement should provide the following information, where applicable:

- Accession codes, unique identifiers, or web links for publicly available datasets
- A list of figures that have associated raw data
- A description of any restrictions on data availability

Genome re-sequencing data have been submitted to NCBI BioProject under accession code PRJNA344883 and PRJNA624267. Other public databases used in this study are OrthoDB (v10;https://www.orthodb.org/) and KEGG PATHWAY Database (Release 91.0;https://www.genome.jp/kegg/pathway.html)

Field-specific reporting

Please select the one below that is the best fit for your research. If you are not sure, read the appropriate sections before making your selection.

☐ Life sciences ☐ Behavioural & social sciences ☒ Ecological, evolutionary & environmental sciences

For a reference copy of the document with all sections, see nature.com/documents/nr-reporting-summary-flat.pdf

Ecological, evolutionary & environmental sciences study design

All studies must disclose on these points even when the disclosure is negative.

Study description	This study includes whole genome sequencing of 180 genomes of Daphnia magna from Belgium. To reconstruct the genomic history, we re-sequenced the 36 D. magna lines resurrected from the OHZ pond and validated it with additional whole genome re-sequencing of 144 D. magna genotypes spread across twelve spatial populations along a fish gradient in the regional ponds.
Research sample	Research samples are composed of Twelve individuals from each subpopulation of Daphnia magna from the sediment core (OHZ pond) and 8-17 individuals per population of Daphnia magna in the spatial survey in Belgium representing fish gradient. A total of 36 genomes (12 genomes per population of the sediment core) were re-sequenced for the resurrection genomic analysis. In addition, the whole genomes of 144 D. magna genotypes representing 12 populations (six populations from ponds without fish (NF) and six populations from ponds with fish (F)) in the region were re-sequenced to allow (a) validation of the findings of the resurrection genomics analysis using six spatial contrasts of NF-F population pairs, and (b) a randomization analysis to establish how many immigrants from the regional genotype pool would be needed to achieve the observed genetic variation in the target population. OHZ_Pre-fish (n=12),OHZ_High-fish (n=12), OHZ_Reduced-fish (n=12), DANA_NF1 (n=11),U2_NF2 (n=12), TER1_NF3 (n=17), MO_NF4 (n=8), KNO15_NF5 (n=10), TER2_NF6 (n=15), ZW4_F1 (n=11), LRV_F2 (n=12), ZW3_F3 (n=14), OHN_F4 (n=12), LRV_F2 (n=12), OM2_F5 (n=10), OM3_F6 (n=12). These samples are sufficient to represent genetic diversity of Daphnia magna in temporal and spatial setting at a regional scale.
Sampling strategy	No sample size selection was performed prior to the study. The dormant stages were hatched in the laboratory and taking advantage of their parthenogenetic reproduction mode as long as conditions are favorable, we started up clonal lines. The resulting clonal lines are each genetically unique, as dormant stages in D. magna are the result of sexual reproduction. The spatio-temporal validation allows robust results.
Data collection	Illumina HiSeq2500 platform (TruSeq SBS Kit v3 reagent kit) for paired-end sequencing with 100 bp read length following manufacturer's instructions at the University of Birmingham, UK under supervision of Dr. Luisa Orsini.
Timing and spatial scale	Laboratory and sequencing works were performed during 2014-2015. Samples were sampled from ponds distributed throughout Belgium. OHZ core was sampled in April 1997 and spatial populations were sampled in the February 2007.
Data exclusions	No data were excluded from the analysis
Reproducibility	The multiple samples from each site representing unique genotype were used in this study.
Randomization	Not applicable
Blinding	No blinding experiments were applicable in this study.

Did the study involve field work? ☐ Yes ☒ No

Reporting for specific materials, systems and methods

We require information from authors about some types of materials, experimental systems and methods used in many studies. Here, indicate whether each material, system or method listed is relevant to your study. If you are not sure if a list item applies to your research, read the appropriate section before selecting a response.

Materials & experimental systems

n/a	Involved in the study
☒	☐ Antibodies
☒	☐ Eukaryotic cell lines
☒	☐ Palaeontology and archaeology
☒	☐ Animals and other organisms
☒	☐ Human research participants
☒	☐ Clinical data
☒	☐ Dual use research of concern

Methods

n/a	Involved in the study
☒	☐ ChIP-seq
☒	☐ Flow cytometry
☒	☐ MRI-based neuroimaging
